# Role of Wild Boar in the Spread of Classical Swine Fever in Japan

**DOI:** 10.3390/pathogens8040206

**Published:** 2019-10-24

**Authors:** Satoshi Ito, Cristina Jurado, Jaime Bosch, Mitsugi Ito, José Manuel Sánchez-Vizcaíno, Norikazu Isoda, Yoshihiro Sakoda

**Affiliations:** 1 Research Center for Zoonosis Control, Hokkaido University, Kita 20, Nishi 10, Kita-ku, Sapporo, Hokkaido 001-0020, Japan; satoshi125@czc.hokudai.ac.jp; 2 VISAVET Center and Animal Health Department, University Complutense of Madrid, 28040 Madrid, Spain; cjdiaz@ucm.es (C.J.); jaimeboschlopez@gmail.com (J.B.); jmvizcaino@ucm.es (J.M.S.-V.); 3 Akabane Animal Clinic, Co. Ltd., 55 Ishizoe, Akabane-cho, Tahara, Aichi-ken 441-3502, Japan; m-ito@oasis.ocn.ne.jp; 4 Global Station for Zoonosis Control, Global Institute for Collaborative Research and Education (GI-CoRE), Hokkaido University, Sapporo 001-0020, Japan; 5 Laboratory of Microbiology, Department of Disease Control, Faculty of Veterinary Medicine, Hokkaido University, Kita 18, Nishi 9, Kita-ku, Sapporo, Hokkaido 060-0018, Japan

**Keywords:** classical swine fever, spatio-temporal analysis, wild boar, transboundary diseases

## Abstract

Since September 2018, nearly 900 notifications of classical swine fever (CSF) have been reported in Gifu Prefecture (Japan) affecting domestic pig and wild boar by the end of August 2019. To determine the epidemiological characteristics of its spread, a spatio-temporal analysis was performed using actual field data on the current epidemic. The spatial study, based on standard deviational ellipses of official CSF notifications, showed that the disease likely spread to the northeast part of the prefecture. A maximum significant spatial association estimated between CSF notifications was 23 km by the multi-distance spatial cluster analysis. A space-time permutation analysis identified two significant clusters with an approximate radius of 12 and 20 km and 124 and 98 days of duration, respectively. When the area of the identified clusters was overlaid on a map of habitat quality, approximately 82% and 75% of CSF notifications, respectively, were found in areas with potential contact between pigs and wild boar. The obtained results provide information on the current CSF epidemic, which is mainly driven by wild boar cases with sporadic outbreaks on domestic pig farms. These findings will help implement control measures in Gifu Prefecture.

## 1. Introduction

Classical swine fever (CSF) is caused by infection with the CSF virus (CSFV), which belongs to the genus *Pestivirus*, family *Flaviviridae*. CSF is described by the World Organisation for Animal Health as a highly contagious febrile disease with potential for high mortality that causes enormous economic loss in the pig industry worldwide [1]. CSFV is a positive-sense, single-stranded RNA virus with a genome of approximately 12.3 kb, comprising one large open reading frame that encodes a polyprotein and flanked by 5’-untranslated region (5’-UTR) and 3’-untranslated region [2]. During virus replication, the polyprotein is processed by cellular and viral proteases into four structural and nine nonstructural proteins [2]. Outbreaks of CSF have been reported over the past decade in Asia (Bhutan, Cambodia, China, India, Indonesia, the Republic of Korea, Lao PDR, Mongolia, Myanmar, Nepal, the Philippines, Thailand, Timor-Leste, and Vietnam), Europe (Latvia, Lithuania, the Russian Federation, Serbia, and Ukraine), Africa (Madagascar), the Caribbean (the Dominican Republic, Guatemala, and Haiti), and Latin America (Bolivia, Colombia, Ecuador, and Peru) [3]. Based on the amino acid sequence of the 5’-UTR and E2, which is one of the structural region of the protein, CSFVs are classified into three genotypes (1, 2, and 3) and several subgenotypes (1.1–1.4, 2.1–2.3, and 3.1–3.4) [4,5]. The virulence of CSFV is categorized via a clinical score into highly virulent, moderately virulent, low virulent, and avirulent [6,7]. Although the CSFV genotype 2.1b isolated from the Republic of Korea was highly virulent, the same genotype isolated in Mongolia was moderately virulent [8,9]. Moreover, the recently classified CSFV genotype 2.1d from China was moderately virulent compared to different variants and antigenicity from field strains identified in China in the past [10]. 

No notifications of CSF were reported in Japan since 1992, and the country had an 11-year stretch of CSF-free status defined by the OIE Terrestrial Animal Health Code since 2007. However, CSF reemerged in Japan in September 2018 in Gifu Prefecture, which is located in the central part of the main island of Japan. Phylogenetic analysis revealed that the CSFV strain isolated in Japan in 2018 showed the highest identity in the complete E2 gene sequence with Chinese strains isolated between 2011 and 2015 and in the partial 5’-UTR sequence with strains isolated in China and Mongolia in 2014 and 2015 [11]. 

By the end of August 2019, a total of 39 CSFV outbreaks on pig farms in four prefectures and 1,071 cases in wild boar in seven prefectures have been reported [12]. Despite the implementation of intensive responses, including movement bans of domestic pigs, surveillance, and oral immunization of wild boar, new notifications of CSF cases in both wild boar and domestic pigs were being reported continually [13]. This might indicate that the pathogenic viruses were widely prevalent and persisted in wildlife around the affected area. As the Eurasian wild boar is also susceptible to CSFV, the circulation and persistence of CSFV among food animals and wildlife makes it difficult to carry out effective control measures for eradicating it in affected areas. Due to contact with infected animals and feeds contaminated with contagious pathogens in garbage dumped on the human sphere, naïve wild boar populations are often infected with CSFV [8,14,15,16,17,18,19,20,21,22,23,24]. Before the 1990s, CSF cases in wild boar were rare concerns as infection was detected rapidly due to the high virulence of circulating strains. However, disease detection appears delayed in the current epidemic due to infection with more moderately virulent strains [25]. As a consequence, there have been serious outbreaks of CSF in the wild boar population in Germany. During an outbreak of CSF in Germany from 1993 to 1998, an epidemiological field investigation confirmed that 59% of the primary cases in domestic pigs could be attributed to either direct or indirect contact with infected wild boar [17]. Virus characteristics and population size can both be considered critical factors for the persistence of CSFV, especially in wild boar populations [25]. It has been suggested that CSFV would be self-limiting within one year in populations of 2000 wild boar, whereas it will persist and become endemic in a larger population [26]. In addition, the population density of wild boar also has been suggested as being a potential factor for the persistence of CSF because more frequent turnover occurs in dense populations, which provides faster renewal of susceptible piglets that increases the chance that the virus will persist in the population [25]. Once the contagious viruses are transmitted to wildlife, specific control measures for wild boar will be needed to eradicate CSF in the affected area and to contain it more effectively. 

The present study conducted a spatio-temporal analysis to obtain epidemiological information on current epidemics of CSF in Japan. Based on the official CSF reports on domestic pig farms and wild boar, notified in Gifu Prefecture from September 2018 to June 2019, we assessed the direction of the spread of the disease and identified areas with high densities of notifications. In addition, to identify spatio-temporal aggregation of notifications and to characterize land cover vegetation in areas of disease aggregation, a clustering analysis was conducted, and obtained clusters were then overlapped with quality habitat map. The obtained information can be used to develop more effective disease control measures for application in both domestic pigs and wild boar. 

## 2. Results

### 2.1. Standard Deviational Ellipse Analysis

**Figure 1:**
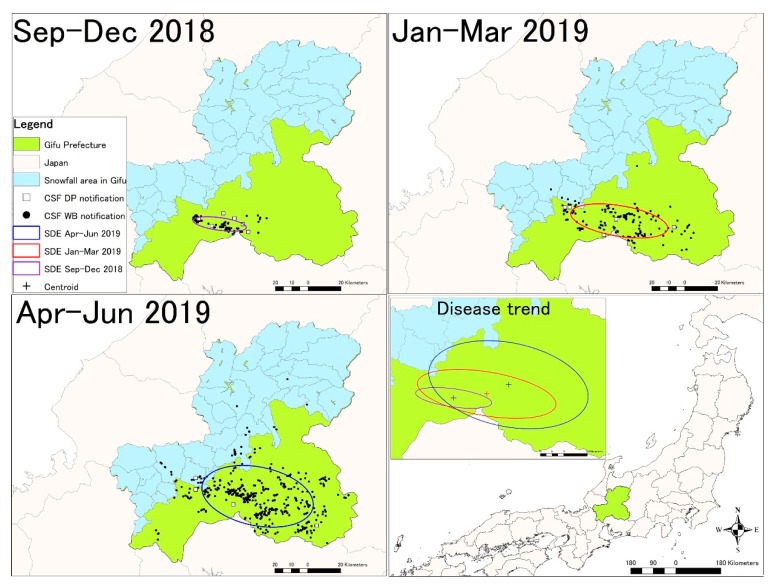
Directional distribution of classical swine fever (CSF) notifications from September 2018 to June 2019. Standard deviational ellipses (SDEs) identified between September and December 2018, between January and March 2019, and between April and June 2019. Ellipses were overlaid with CSF notifications distinguishing domestic pig (DP) (square) and wild boar (WB) (circle). Ellipses with centroids were combined to indicate the directional trend of the CSF outbreaks.

A standard deviational ellipse analysis was applied to describe the directional trend and dispersion of CSF notifications in the study area throughout the study period. The study covered the period between September 2018 and June 2019, which was divided into three stages (September–December, January–March, and April–June). Figure 1 illustrates standard deviational ellipses and CSF notifications between September 2018 and June 2019 (Figure 1). To indicate the potential explanation for the directional trend of the CSF outbreaks, the ellipses were overlaid on a map of snowfall area in Gifu Prefecture obtained from the National Land Information Division, Ministry of Land, Infrastructure, Transport and Tourism [27]. The findings showed that CSF notifications appeared to move northeast while spreading along the border of the snowfall area. 

### 2.2. Multi-Distance Spatial Cluster Analysis

The multi-distance spatial cluster analysis was applied to explore the maximum distance between cases of CSF notifications. The results indicated that 23 km was the maximum distance of the significant spatial association between CSF notifications in Gifu Prefecture. The obtained maximum distance was used in the subsequent analyses.

### 2.3. Kernel Density Estimation Analysis

**Figure 2:**
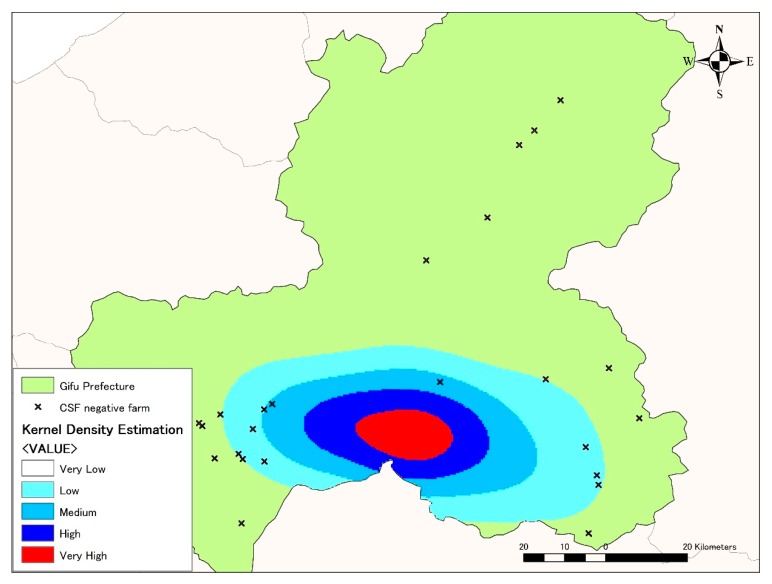
Density of CSF notifications in Gifu Prefecture. The heat map illustrates the estimated kernel density of CSF notifications (notifications/km^2^) from very high (red) to very low (transparent). Each coloured area indicates the density of CSF notifications per square kilometer: very high (>0.400), high (0.300–0.399), medium (0.200–0.299), low (0.100–0.199), and very low (<0.100). The highest density of CSF notifications was located in the southern part of Gifu Prefecture. A very low density of CSF notifications was located in other areas of the prefecture. Locations of pig farms not affected by CSF are represented by crosses.

The kernel density estimation analysis was applied to describe the spatial distribution of the CSF notifications. The analysis showed that the highest density of CSF notifications was located in the southern part of Gifu Prefecture (Figure 2) with further expansion to the east. Among the 16 CSF-positive farms, 37.5% were located in areas with very high or high density of notifications, 31.25% in areas of medium density and 31.25% in areas of low density. Moreover, most of the non-affected domestic farms were located in areas with very low density of notifications (80%), followed by areas with low density (20%). The analysis revealed that CSF-positive farms were located in areas with higher density of notifications, whereas the non-affected farms tended to locate in areas with low density.

### 2.4. Space-Time Cluster Analysis

The space-time permutation analysis was applied to analyze the space-time patterns of the CSF notifications. The analysis identified two significant space-time clusters (*P* < 0.05) in Gifu Prefecture during the study period. Cluster 1, which had a radius of 12.12 km, covered 9 September 2018 to 13 January 2019, and contained 83 notifications, including 4 outbreaks on domestic pig farms. Cluster 2 had a radius of 19.79 km, spanning the period from 11 February 2019 to 19 May 2019, and contained 198 notifications, including three outbreaks in domestic pigs (Table 1). 

### 2.5. Quality of Available Habitat (QAH) Within Space-Time Cluster Area 

**Figure 3:**
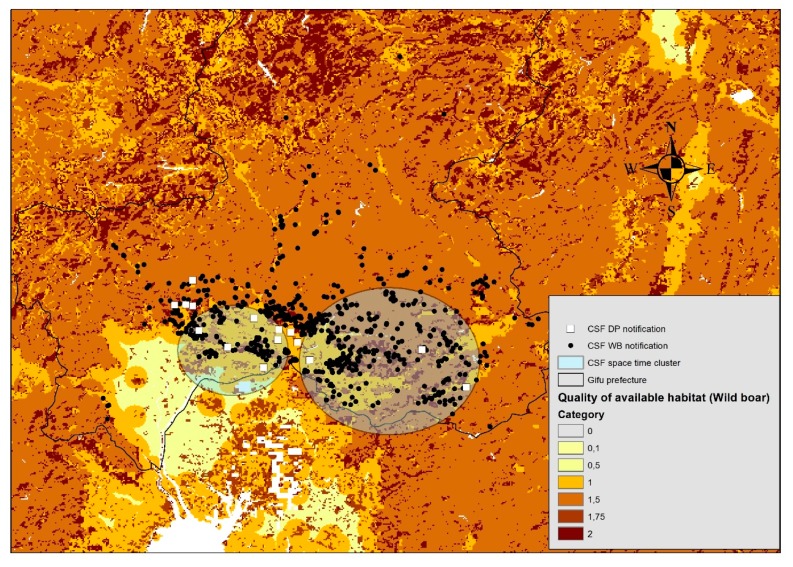
Locations of the significant space-time clusters of CSF. Notifications: (*P* < 0.05) in Gifu Prefecture overlaid on a map of the quality of available habitat (QAH) levels for wild boar. Graduated colors indicate the quality of habitat availability from darker colors (areas with better quality of habitat availability) to lighter colors (areas with worse quality of habitat availability).

In order to characterize the land cover vegetation within two significant space-time clusters, the clusters were overlaid with a QAH map. The results showed different patterns between cluster 1 and cluster 2 (Figure 3). In cluster 1, 50.6% of CSF notifications were reported in areas at QAH 1, while 31.3% were reported in areas at QAH 1.5, and 18.1% were reported in areas at QAH 2 (Table 2). In cluster 2, 22.7% of CSF notifications were reported in areas at QAH 1, 52.5% were reported in areas at QAH 1.5, 2.5% were reported in areas at QAH 1.75, and 22.2% were reported in areas at QAH 2 (Table 2). 

The CSF notifications within clusters 1 and 2 occurred within habitats that included rainfed croplands (QAH 1), a closed (>40%) needle-leaved evergreen forest (>5 m) (QAH 1.5), a mosaic of cropland (50%–70%) and vegetation (grassland/shrubland/forest) (20%–50%) (QAH 1.75), a mosaic of vegetation (grassland/shrubland/forest) (50%–70%) and cropland (20%–50%) (QAH 2), closed (>40%) broadleaved deciduous forest (>5 m) (QAH 2), and closed to open (>15%) mixed broadleaved and needle-leaved forest (>5 m) (QAH 2).

Although different patterns of land cover vegetation were observed between clusters 1 and 2, nearly 50% of CSF notifications within cluster 1 and more than 75% within cluster 2 were notified in QAH 1.5–2, which provides the greatest opportunities for food and shelter for wild boar. 

## 3. Discussion

From 2018 until August 2019, all notifications of CSF outbreaks in Japan have been made in Gifu Prefecture as well as in the surrounding four prefectures. A total of 1110 notifications had been reported so far, with 1071 affecting wild boar and 39 affecting domestic pig farms. The continuous notification of CSF in the area might have been attributed to wide spread of the virus within wild boar populations favored by free animal movements, as well as to the emergence of epidemiologically related domestic pig farms. To prevent the disease spreading in wild boar, control measures including (i) fencing to restrict animal movements, (ii) hunting activities for active monitoring and to reduce susceptible populations, and (iii) disseminating baits for oral immunization, were implemented. However, the efficacy of these strategies has not been confirmed. Therefore, we conducted a spatio-temporal analysis to obtain epidemiological information of the spread of CSF in Gifu Prefecture. Results from this analysis could help to increase our understanding of the current CSF epidemic and to contribute strategies for the containment of the disease in domestic pigs and wild boar. 

Japan is an island country that has achieved the status of freedom from several contagious animal diseases by implementing adequate control measures that take advantage of the country’s geography. Nevertheless, Japan has imported outbreaks of contagious animal diseases from neighboring countries. In 2010, there was an outbreak of foot-and-mouth disease (FMD) in Miyazaki Prefecture in the southern part of Japan, which caused extensive losses in animal husbandry. According to the high degree of sequence homology between an original virus isolated in Japan and viruses that were circulating widely in East Asia, it was suspected that the FMD virus might have been introduced via movement of people or commodities from East Asia [28]. The high homology of genetic sequences between the CSF virus isolated in Japan and viruses prevailing in China suggests that the infectious CSF virus may have been introduced from China. Potential factors that could have contributed to disease introduction include easy access from the international airport to the affected area, which has regular and direct flights from China, and the relatively high population density of Chinese people in the affected area. 

In the present study, standard deviational ellipse analysis was conducted to measure the standard distance of CSF notifications. Shifting the centroids of identified ellipses indicated that the disease notification has spread in a northeast direction. Overlaying the three identified ellipses with a map of snowfall area in Gifu Prefecture revealed that the disease spread along the border of the snowfall area. In the south of Gifu Prefecture, there is a widespread area of flat land with field crops or animal farms, residential areas, and forests surrounded by mountains to the north. As suggested by other authors [29,30], wild boar do not move to the snowfall or high mountain areas. Therefore, mountains could have acted as an effective geographical barrier to limit wild boar movements and guide the direction of the spread of CSF. 

Another concern regarding the spread of the disease is the potential for it to jump to remote areas. During the epidemic, CSFV infections were confirmed on seven farms that were geographically distant from, but epidemiologically linked, to the farms affected by CSFV (i.e., run by the same owner, supported by the same husbandry company, etc.) [13]. Given the potential for transmission of the virus between pigs on any farms or from wild boar near that farm, the epidemiologically related farms may further expand the spread of disease. This “disordered” spread of disease could affect the accuracy of spatio-temporal analysis by overestimating the maximum distance of significant spatial association between notifications. During the FMD epidemic in Miyazaki, the disease was confirmed 70 km away from the zone of movement restriction, which could have been caused by vehicle transportation [28]. Unexpected occurrences of disease in epidemiologically related farms would require reviewing farm biosecurity measures, as well as disease monitoring protocols. 

In the present study, the results of the multi-distance spatial cluster analysis revealed that the maximum distance of relationship between CSF notifications was 23 km. Because of the small number of CSF outbreaks on domestic pig farms, we estimated the maximum distance of the relationship between notifications of domestic pigs and wild boar. This assumption could have influenced our estimated distance resulting in overestimation due to long distance spread observed on domestic pig farms. Nevertheless, similar approaches have studied another transboundary animal disease, African swine fever (ASF), which shares hosts and most of the transmission mechanisms with CSF [31,32,33]. When comparing our results with other studies, the estimated distance (23 km) was similar to that obtained for notifications of ASF in domestic pigs (15 km) and wild boar (25 km) in Sardinia [32]. This finding may be useful for setting the range of effective surveillance and control zones in the affected area.

The application of cluster analysis to identify areas with significant spatio-temporal aggregation of the ASF outbreaks in Sardinia from 2004 to 2013 indicated four clusters, the largest of which had a radius of 30 km [33]. This does not correspond with the results of another report that identified one cluster with a radius of 3 km in the same area [32]. As discussed in Iglesias et al., methodological differences could have led to the discrepancy [32]. In present study, because of the small number of CSF outbreaks in pig farms, we could not identify the maximum distance for the relationship between notifications of CSF in pigs alone, but we were able to do it by considering pigs and wild boar together. The discordance between the findings of the two spatio-temporal analyses in Sardinia may suggest that by using mixed data for two species in the present study, we may have overestimated the distance of the spread of disease compared to true distance of transmission in each of the two species. However, we believe that this uncertainty would be acceptable for setting the monitoring area with high efficacy. Thus, these findings may be useful for setting the range of an effective surveillance and control zone.

Data on wild boar cases consisted of animals found dead and/or captured during surveillance activities. Many of wild boar were captured during active surveillance activities by setting traps and conducting hunting activities. Considering that most of the reported wild boar cases were located close to human habitats, the wild boar capture area may have been biased. Therefore, the disease could be wider spread in the area than what has been reported in official notifications, and the identified clusters could have had a shorter radius. Ideally, active virologic surveys should be intensively implemented to decrease the reporting biases by providing more samples to detect low levels of prevalence [34,35]. The Gifu Animal Health Administration has authorized hunting activities to reduce the number of susceptible, as well as potentially infected, individuals. Hunters are a critical group for implementing population control and proper disposal of wild boar carcasses. 

According to the investigative report of the affected farms, there were some factors that might have increased the risk of CSFV introduction into affected farms, including (i) improper preparedness against invasion of wild or small animals into farms; (ii) imperfect clothing and boot changes in farms and pig pens, or disinfection of those materials; and (iii) inadequate vehicle disinfection [13]. To prevent contact among each of the hosts, in addition to raising awareness of disease among farmers and hunters, it is important to improve biosecurity measures in pig farms against CSFV as well as other infectious diseases. 

Finally, we analyzed the QAH level of areas within the two identified clusters to characterize land cover vegetation in areas of disease aggregation. According to Bosch et al., a QAH 1 level corresponded to suitable areas for food or shelter for wild boar (mainly agricultural landscapes) [36]. In cluster 1, 50.6% of CSF notifications were reported in areas at QAH 1, whereas in cluster 2, 22.7% of CSF notifications were reported in areas at QAH 1. Considering that frequent direct and indirect contact is likely to occur between both hosts, contagious viruses in wild boar could be transmitted to pigs in the farms due to insufficient biosecurity in the affected farms since wild boar was the suspected source of infection on 80% of affected domestic pig farms in Gifu Prefecture during the studied epidemic [13,35]. On the other hand, almost 50% of CSF notifications within cluster 1 and over 75% within cluster 2 were associated with QAH 1.5–2, which mainly corresponded to natural landscapes. These natural areas provided the greatest opportunities for food and shelter for wild boar. In the case of ASF, it has been reported that wild boar can transmit the disease efficiently at local levels within their own population [32,36]. Furthermore, De la Torre et al. suggested that the spread of ASF in Europe was driven by contact between animals from different populations that moved short distances [37]. Although ASF is caused by another virus, given that wild boar play an important role in both diseases, it is plausible to assume that CSF also could have expanded through contact between individual wild boar. Therefore, it would be critical to control wild boar populations and manage wild boar carcasses adequately from the environment to reduce habitat contamination. 

Interestingly, the QAH map could also identify routes of CSF introduction or spread, mediated by wild boar, through vegetation or travel corridors. Travel corridors are either unbroken vegetation corridors or patches of habitat that enable animals to travel securely from one habitat to another [36]. These patches of habitat and vegetation corridors could be used as strategic points of vaccination where oral baits could be placed. In Gifu Prefecture, the vegetation is composed mainly of broadleaved evergreen and broadleaved deciduous forests, which provide suitable habitat for wild boar [38,39]. Given that the composition of the vegetation in Gifu Prefecture is common throughout Japan, it is likely that the disease could spread similarly to other prefectures.

It should be noted that vegetation types and wild boar behavior could vary among geographical features. For example, mountains usually have gentle slopes in Germany, whereas Japanese mountains tend to have precipitous slopes [40]. These topographical differences may require different approaches for control of wild boar populations.

Almost one year has passed since the first notification of the CSF outbreak in Japan, and the spread of the disease has been confirmed mainly in wild boar. Fortunately, CSF outbreaks on domestic pig farms have been limited. Nevertheless, the potential risk of CSF introduction on farms could be high due to limited biosecurity, high number of wild boar cases in the area, and difficulties in implementing disease control measures in wildlife [13]. The results from this study provide information on the current epidemic, which may help improve current approaches for controlling CSF in Japan. Information on the direction and distance of disease spread could help with the implementation of control measures by modifying the area for control and surveillance zones or identifying specific locations for increasing efforts of oral immunization. 

Given the potential risk of the ASF introduction from neighboring countries, we should summarize and disseminate the lessons learned from the current CSF outbreak to achieve the protection of ASF invasion or rapid containment of its occurrence even if it occurred.

## 4. Material and Methods

### 4.1. Data and Data Sources

Epidemiological data for the periods from 9 September 2018 to 25 June 2019 were provided by the Gifu Prefectural Government, which provided the dates and coordinates (latitude and longitude) of the notifications of CSF in domestic pigs and wild boar. A total of 743 CSF notifications, 16 outbreaks on domestic pig farms, and 727 cases in wild boar were confirmed by RT-PCR and/or ELISA tests in the laboratory [13]. As we focused on local transmission of CSFV, notifications of CSF in slaughterhouses or in facilities through which CSF-affected pigs had been transported were removed from the current study. Notifications of CSF in wild boar reported on the same day and location were regarded as one case.

### 4.2. Standard Deviational Ellipse Analysis

Standard deviational ellipse (SDE) analysis is a tool that provides the orientation and shape of a distribution, as well as its location, and dispersion or concentration of the data [41]. It requires a single point that is used to define the standard deviational ellipse. The analysis was conducted to describe the trend and spatial characteristics of CSF notifications in the study area in ArcGIS 10.6.1 software (ESRI Inc., Redlands, CA, USA) following an approach similar to Fonseca et al. and Lu et al. [42,43]. The ratio (R) of the long and short axes was used to identify the degree of clustering (R > 1) or dispersion (R = 1) [42,43]. To analyze temporal changes of CSF notifications, the study period was divided into three stages—(i) September to December 2018 (four months), (ii) January to March 2019 (three months), and (iii) April to June 2019 (three months).

### 4.3. Multi-Distance Spatial Cluster Analysis

A multi-distance spatial cluster analysis tool in ArcGIS software version 10.6.1 was used to identify the maximum distance of the relationships between CSF notifications according to the guide on the manufacture’s website [44]. In brief, the tool uses a common transformation of Ripley’s k function, wherein the expected result with a random set of events is equal to the input distance. The transformation L(d) is given by the following formula:

$$L\left(d\right)=\sqrt{\frac{A\sum_{i=1}^{N}\sum_{j=1,j\neq 1}^{N}k\left(i,j\right)}{\pi N\left(N-1\right)}}$$

where *A* is the area, *N* is the number of events, *d* is the distance, and *k*(*i*, *j*) is the weight, in which it is 1 when the distance between *i* and *j* is less than or equal to d and it is 0 when the distance between *i* and *j* is greater than *d*. To analyze the spatial pattern of CSF notifications, Observed K values were compared to the Expected K values of a completely random spatial distribution of CSF notifications with 999 simulations, which is equal to confidence levels of 99.9%. 

The Diff K values contain the Observed K values minus the Expected K values. In the present analysis, the Expected K values that yield the highest Diff K values were applied as the maximum distance for relationships between notifications of CSF outbreaks in Gifu Prefecture.

### 4.4. Kernel Density Estimation Analysis

Kernel density estimation is a non-parametric estimator for describing the spatial extent of a series of events [45]. In the current study, the kernel density tool was applied to explore the influence of the CSF notifications in the study area by calculating the density of CSF notifications in ArcGIS 10.6.1. A radius of 23 km based on results obtained from Ripley’s k function, was applied as the maximum distance for significant spatial association between CSF notifications. Kernel density estimation was divided into five categories according to the equal interval method. 

### 4.5. Space-Time Cluster Analysis

A space-time permutation technique was applied to examine the presence of space-time clusters in Gifu Prefecture. The upper limit on the geographical size of the cluster was set as 23 km, the minimum time aggregation as seven days, and the maximum temporal cluster size as 50% of the total study period (default setting) [32]. A Monte Carlo process was implemented using 999 replications to test for the presence of candidate clusters (*P* < 0.05). Analyses were conducted in SaTScan software v9.6 (Kulldorff, Boston, MA, USA) [46].

### 4.6. QAH Within Space-Time Cluster Area

CSF notifications within significant space-time clusters were overlaid on a QAH map to characterize land cover vegetation in areas of disease aggregation. The QAH map developed by Bosch et al. [36] is a cartographic tool previously suggested as a potential tool for managing African swine fever. Briefly, it is a standardized distribution map based on global land cover vegetation (GLOBCOVER) that quantifies QAH for wild boar [47]. The QAH map provides seven levels of QAH, namely (i) 0, “absent”; (ii) 0.1, “unsuitable”; (iii) 0.5, “worst suitable area”; (iv) 1, “suitable areas for food or shelter”; (v) 1.5, “suitable areas for food and shelter, but used mainly for one or the other”; (vi) 1.75, “suitable areas for food and shelter, but mainly used for food”; and (vii) 2, “suitable areas for both food and shelter.” In addition, the QAH map also differentiates between landscapes such as natural (mainly QAHs 2 and 1.5) and agricultural landscapes (QAHs 1.75 and 1), among others. 

## Figures and Tables

**Table 1:** Observed and expected notifications, duration, start and end dates, and radius of each space-time cluster detected (*P* < 0.05) in CSF notifications in Gifu Prefecture.

Cluster	Observed Notifications	Expected Notifications	Duration (Days)	Start Date	End Date	Radius (km)
1	83	17.34	124	2018/9/9	2019/1/13	12.12
2	198	131.87	98	2019/2/11	2019/5/19	19.79

**Table 2:** Quality of availability habitats (QAH) of CSF notifications within the two identified space-time clusters.

QAH Category	Land Cover	Cluster 1			Cluster 2		
		DP (*n*)	WB (*n*)	Total (*n*) (*%*)	DP (*n*)	WB (*n*)	Total (*n*) (*%*)
1.0	Rainfed croplands	4	38	42 (50.6)	0	45	45 (22.7)
1.5	Closed (>40%) needleleaved evergreen forest (>5m)	0	26	26 (31.3)	3	101	104 (52.5)
1.75	Mosaic cropland (50–70%)/vegetation (grassland/shrubland/forest) (20–50%)	0	0	0 (0.0)	0	5	5 (2.5)
2.0	Mosaic vegetation (grassland/shrubland/forest) (50–70%)/cropland (20–50%)	0	0	0 (0.0)	0	14	14 (7.1)
2.0	Closed (>40%) broadleaved deciduous forest (>5m)	0	1	1 (1.2)	0	0	0 (0.0)
2.0	Closed to open (>15%) mixed broadleaved and needleleaved forest (>5m)	0	14	14 (16.9)	0	30	30 (15.2)
	Total	4	79	83 (100.0)		195	198 (100.0)

DP: domestic pig. WB: wild boar. *n*: the number of notifications.

## Founding

This work was supported in part by a Grant-in-Aid for Scientific Research (B) (JSPS KAKENHI Grant Number 19H03115) from the Japan Society for the Promotion of Science.
